# The Cerebellum Predicts the Temporal Consequences of Observed Motor Acts

**DOI:** 10.1371/journal.pone.0116607

**Published:** 2015-02-17

**Authors:** Laura Avanzino, Marco Bove, Elisa Pelosin, Carla Ogliastro, Giovanna Lagravinese, Davide Martino

**Affiliations:** 1 Department of Experimental Medicine, Section of Human Physiology, University of Genoa, Genoa, Italy; 2 Department of Neuroscience (DiNOGMI), University of Genoa, Genoa, Italy; 3 Neurology Department, King's College Hospital, London, United Kingdom; 4 Queen Elizabeth Hospital, Woolwich, London, United Kingdom; 5 Centre for Neuroscience and Trauma, Queen Mary University of London, London, United Kingdom; Duke University, UNITED STATES

## Abstract

It is increasingly clear that we extract patterns of temporal regularity between events to optimize information processing. The ability to extract temporal patterns and regularity of events is referred as temporal expectation. Temporal expectation activates the same cerebral network usually engaged in action selection, comprising cerebellum. However, it is unclear whether the cerebellum is directly involved in temporal expectation, when timing information is processed to make predictions on the outcome of a motor act. Healthy volunteers received one session of either active (inhibitory, 1Hz) or sham repetitive transcranial magnetic stimulation covering the right lateral cerebellum prior the execution of a temporal expectation task. Subjects were asked to predict the end of a visually perceived human body motion (right hand handwriting) and of an inanimate object motion (a moving circle reaching a target). Videos representing movements were shown in full; the actual tasks consisted of watching the same videos, but interrupted after a variable interval from its onset by a dark interval of variable duration. During the ‘dark’ interval, subjects were asked to indicate when the movement represented in the video reached its end by clicking on the spacebar of the keyboard. Performance on the timing task was analyzed measuring the absolute value of timing error, the coefficient of variability and the percentage of anticipation responses. The active group exhibited greater absolute timing error compared with the sham group only in the human body motion task. Our findings suggest that the cerebellum is engaged in cognitive and perceptual domains that are strictly connected to motor control.

## Introduction

The traditional view of the cerebellum as a structure exclusively engaged in motor control has been largely modified in recent years, with increasing evidence pointing towards an involvement of cerebellar circuits in several domains pertaining to cognition and emotion [1–4]. Among cognitive and perceptual activities, the cerebellum seems strongly implicated in temporal processing [5,6]. Timing is a fundamental feature of human movement, perception and cognition. Sensory events may have temporal lengths or they may define boundaries of “empty” temporal intervals. Likewise, moving targets possess temporal properties that need to be identified in order to assess their future trajectories [7]. In action, timing is essential when producing sequences and when coordinating our movements to those of various moving objects in the external environment [5]. Indeed, the processing of time-dependent features of movement has a crucial role in predicting whether the outcome of a complex motor sequence, such as handwriting or playing a musical passage, will be consistent with its ultimate goal, or results instead in an execution error. Further, it is increasingly clear that, to optimize information processing, we extract patterns of temporal regularity between events. The ability to extract temporal patterns and regularity of events is referred as temporal expectation [8]. Recently we developed a novel paradigm to test the ability to correctly make predictions about the temporal characteristics of a writing movement in a group of patients with writer’s cramp [9]. We found an abnormal timing of visually perceived motion, assessed through our temporal expectation task, in patients with writer’s cramp, likely for an abnormality in the integrative role of the cerebellum over sensory and motor cortical areas while the subject was structuring a mental representation of the handwriting motor sequence. This hypothesis was driven by the fact that temporal expectation activates the same cerebral network usually engaged in action selection, comprising cerebellum [10–12] together with the notion that the cerebellum is likely to be a component of the cerebral network responsible of dystonia pathophysiology [13,14].

However, it is still an open question whether the cerebellum might be involved in temporal expectation, when timing information is processed to make predictions on the outcome of a motor act, in the absence of self-action. By applying a conditioning rTMS train on cerebellar regions, it is possible to induce a transient modulation of the activity of the cerebellar cortex, hence to investigate whether changes in cerebellar neural activity would interfere with a specific task. Inhibitory rTMS (1Hz-rTMS) over lateral cerebellum has been used to explore the role of cerebellum in motor timing [15–17].

As in our previous study [9] healthy volunteers were asked to temporally predict, with a keyboard click, the end of a human movement (handwriting) or of an inanimate object (a moving circle reaching a spatial target) perceived on a computer screen. One group of participants underwent inhibitory 1Hz-rTMS over the right lateral cerebellum, whereas a second group received sham stimulation. We hypothesized a direct role of the cerebellum in temporal expectation when the aim of the task was that of predicting the outcome of a human movement.

## Material and Methods

Twenty-six subjects without any history of neurological or psychiatric disease (13 females and 13 males) were recruited for the study and divided in two age- and gender-matched groups. The first group (1Hz-rTMS, n = 12; mean age = 25.6±3.6; 6 males) performed the perceptual temporal expectation task immediately after receiving an active inhibitory rTMS train of stimuli on the right lateral cerebellum, whereas the second group (Sham, n = 14; mean age = 24.8±4.1; 7 males) performed the task immediately after receiving sham rTMS stimulation on the same area. Importantly, a between-subjects design was preferred to a within-subjects design, in order to avoid a learning effect on the task performance. All subjects were right-handed. All participants gave their written informed consent prior to their inclusion in this study. The experimental protocol was approved by the ethics committee of the University of Genoa and was carried out in agreement with legal requirements and international norms (Declaration of Helsinki, 1964).

### Temporal expectation task

The experimental paradigm consisted of one session assessing temporal expectation of human body segment versus inanimate object motion. This task has been described elsewhere [9]. Briefly, it consisted of two different perceptual tasks: a target task, involving the perception of a common movement of a human body segment (a right hand writing a sentence), and a control task, involving the perception of a movement of an inanimate object (a ball reaching a target). These movements were presented as video files on a computer screen at a fixed speed. The human body motion video displayed the movement of a hand writing a proverb sentence in Italian (‘il mattino ha l’oro in bocca’) with a black pencil on an A4-size white paper sheet positioned on the table; the inanimate object motion video consisted of a linear movement, directed from left to right, of a black ball on a white screen heading towards a black vertical bar. The two videos displayed movements under the same visual angle, had the same total duration (20 s), and the order of administration was counterbalanced across the two groups. The tasks have been programmed using dedicated software (E-Prime 2.0, SciencePlus). For each of the two tasks, subjects were shown the full video for a single time without receiving any instruction. The actual task consisted of watching the same video, but it was interrupted after a variable interval from its onset (pre-dark interval) by a dark interval of variable duration (dark interval). During the dark interval, subjects were asked to indicate when the movement represented in the video reached its end by clicking on the space bar of the keyboard.

**Fig 1:**
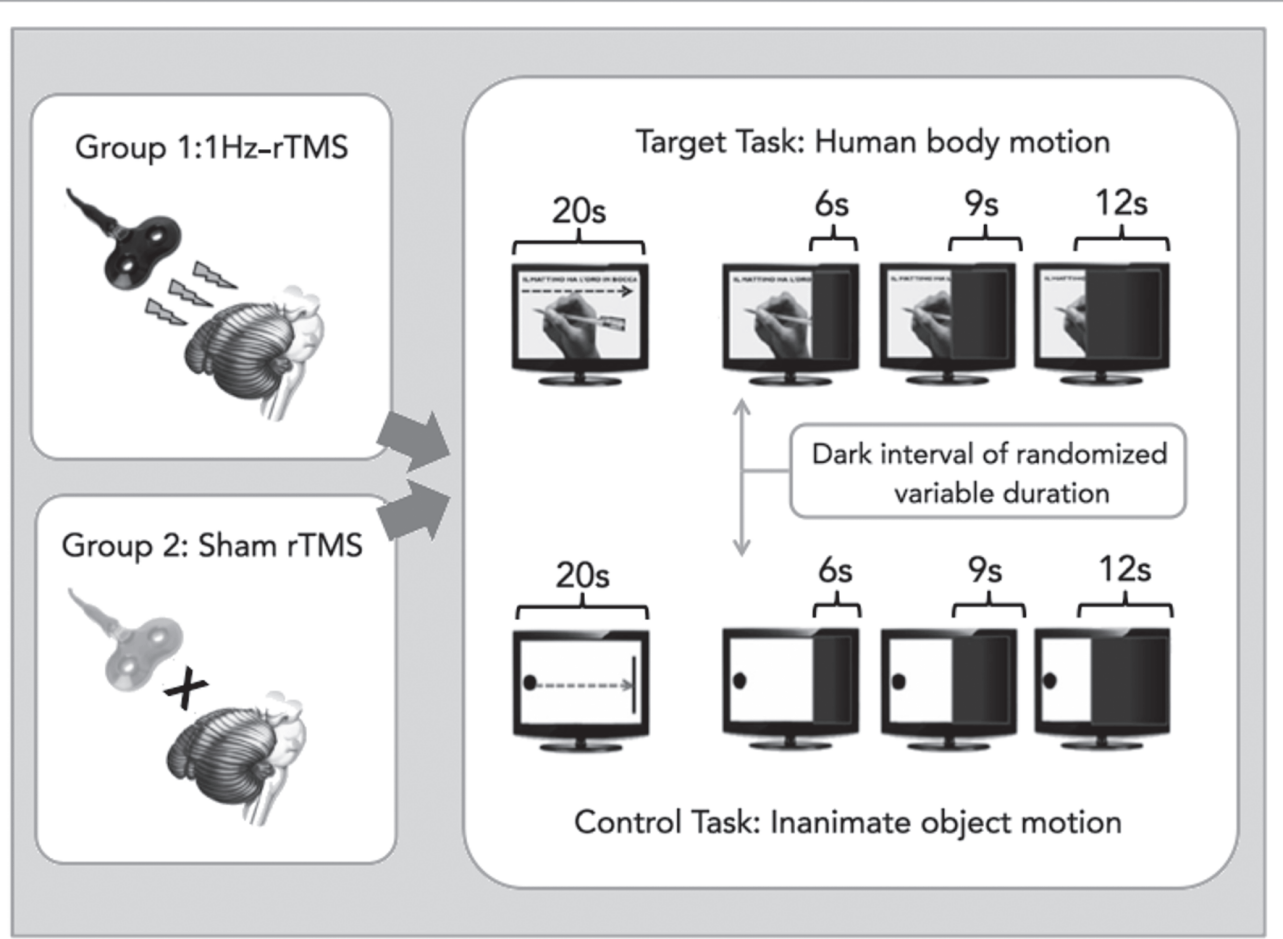
A schematic view of the experimental design. Healthy volunteers received one session of either active, inhibitory, 1Hz (Group1) or sham (Group2) repetitive transcranial magnetic stimulation (rTMS) covering the right lateral cerebellum prior the execution of a temporal expectation task. Two videos were shown once to the subjects: “writing” video showed a common movement of a human body segment (a right hand writing a sentence), while “ball” video showed a movement of an inanimate object (a ball reaching a target). The task consisted in watching the same video, but interrupted, after a variable interval from its onset by a dark interval of variable duration (6, 9 and 12 seconds). During the *dark* interval subjects were required to judge the duration of video clicking on space bar when they reckoned that the movement had reached its end.

Thus, participants used their knowledge of velocity of motion to extrapolate duration of the movement of the body segment (or object) represented in the video. For the sake of the analysis, we refer to the period spanning from the beginning of the dark interval to the subject’s response as ‘reproduced interval’, whereas we refer to the real duration of the dark interval as ‘target interval’. Participants did not receive any feedback on interval duration or their performance throughout the whole session. Three different dark intervals (6, 9 and 12 s) were used. Separately for each task (*target and control task*), each condition (6, 9 and 12 s dark intervals) was administered six times in a randomized order, for 36 trials per experimental session. Handwriting (*target task*) and inanimate object motion (*control task*) were presented in separate blocks; the order of presentation of the blocks was random across subjects. The experimental design is summarised in Fig. 1.

### rTMS procedure

TMS was performed with a Magstim Rapid magnetic stimulator (Magstim Company, Whitland, Dyfed, UK) connected to a figure-of-eight coil (wing diameters of 90 mm) positioned over the right lateral cerebellum (3 cm right and 1 cm inferior to the inion). The coil was positioned tangentially to the scalp with the handle pointing up. The current in the coil was directed upwards, which induced a downward current in the cerebellar cortex [18]. The scalp coordinates were the same adopted in previous studies, in which MRI reconstruction and neuro-navigation systems showed that cerebellar TMS in this site predominantly target the posterior and superior lobules of the lateral cerebellum [15,16]. This approach has been adopted in previous investigations in which cerebellar rTMS was shown to be effective in modulating the excitability of the contralateral motor cortex [19,20] and interfering with cognitive functions such as procedural learning and sub-second time perception [21,22]. The exact coil position was marked by an inking pen to ensure an accurate positioning of the coil throughout the experiment. The stimulating coil was held by hand and coil position was continuously monitored throughout the experiment. Stimulation of structures deeper than the cortical cerebellar layers seems unlikely, owing to the fact that the effects of the magnetic field do not go beyond 2–3 cm below the scalp [18].

A single train (duration: 10 minutes; 600 stimuli) of 1Hz-rTMS was delivered at 90% of the motor threshold (MT) on right cerebellum. For sham rTMS the coil was positioned over the same scalp site, but angled away (90-degrees) so that no current was induced in the brain.

The MT was determined as the minimum stimulus intensity capable of eliciting a motor evoked potential (MEP) of at least 50 μV in the contralateral first dorsal interosseus (FDI) muscle in five or more of ten consecutive stimulations. EMG was recorded with silver disc surface electrodes placed in a tendon belly arrangement over the FDI muscle. The ground electrode was placed at the wrist. EMG signals were amplified and filtered (20 Hz to 1 kHz) with a D360 amplifier (Digitimer Limited, Welwyn Garden City, UK). The signals were sampled at 5000 Hz, digitized using a laboratory interface (Power1401, Cambridge Electronics Design, CED, Cambridge, UK) and stored on a personal computer for display and later off-line data analysis.

### Data analysis

Performance on the temporal expectation task was analysed measuring the timing error, the normalized absolute value of timing error, the coefficient of variability and the percentage of anticipation responses. These parameters allowed us to describe the ability in time processing determining the magnitude (absolute value of timing error) and the variability (coefficient of variability) of the timing error, as well as the strategy adopted by subjects during time prediction (timing error and percentage of anticipation responses).

The timing error was defined as the time elapsing between the subject’s response and the real end of the video, and had a negative value when the response preceded the real end of the video, and positive when the response was delayed in respect to the real end of the video. The absolute value of this error was then normalized with respect to the corresponding target interval and was expressed as a percentage. This parameter provides a measure of the accuracy of subjects in estimating the corresponding target interval: the greater the absolute error, the greater the error in temporal judgement regardless of its direction.

The coefficient of variability was calculated as Standard Deviation (SD)/mean of the reproduced intervals, representing an index of performance variability in temporal judgement. Finally, the percentage of anticipation responses (calculated on the total number of responses) represents the number of responses in which subjects pressed the space button before the real end of the movement (negative error). Together with the timing error, this parameter gives information on the tendency to under- or over-estimate the target interval.

### Statistical analysis

Timing parameters (timing error, absolute value of timing error, coefficient of variability and percentage of responses in anticipation) were analysed by means of a repeated measures ANOVA with group (1Hz-rTMS and Sham) as between subject factor and task (target, control) and target interval (6, 9 and 12 s) as within- subjects factors. Post hoc analysis of significant interactions was performed by means of t-tests applying the Bonferroni correction for multiple comparisons where necessary. P-values of < 0.05 were considered as threshold for statistical significance. Statistical analysis was performed with SPSS 13.0.

## Results

The performance of the temporal expectation task was influenced by the active 1Hz-rTMS on right lateral cerebellum, as evidenced by significant changes in the absolute timing error parameter.

**Fig 2:**
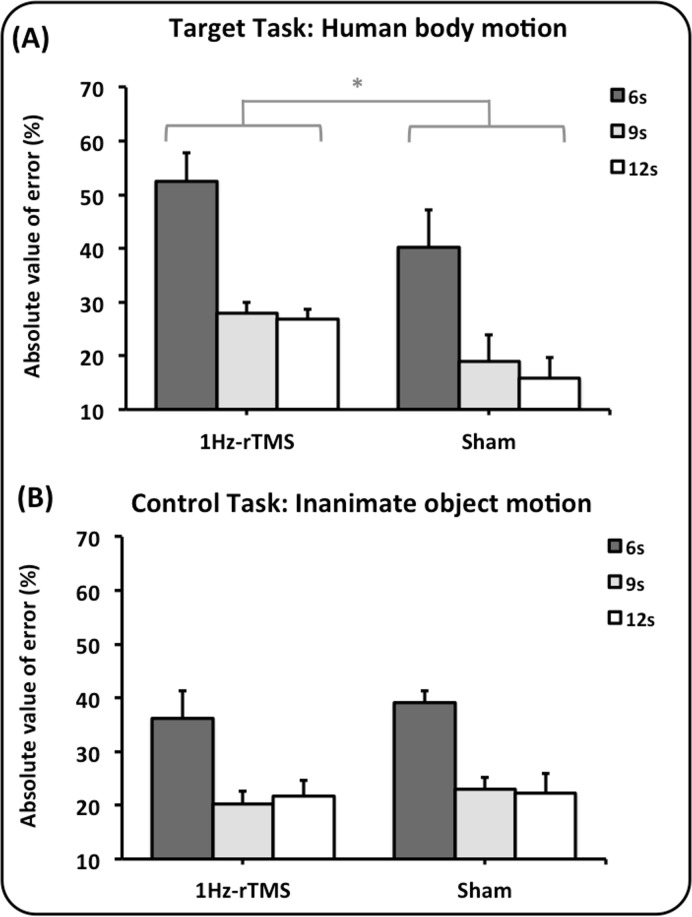
Effect of 1Hz-rTMS and Sham rTMS on lateral cerebellum on the absolute value of timing error. Abscissa: the type of conditioning stimulation (1Hz-rTMS and Sham). Ordinate: the duration of the absolute value of error expressed as percentage of the duration the target interval. Panel A depicts the results of the human body motion perceptual task, whereas panel B depicts the results of the inanimate object motion task. Colours code the duration of the dark interval: dark grey, 6 seconds; light grey, 9 seconds; white, 12 seconds. Mean data + standard error mean (SEM) are shown. Asterisks indicate that the absolute timing error, at all target intervals, was significant larger in the human body motion task (handwriting) with respect to sham (*p<0.05).”

In the presence of 1Hz-rTMS on the lateral right cerebellum, participants exhibited a greater normalized absolute timing error, at all target intervals in the human body motion task (handwriting) with respect to sham (Fig. 2). Accordingly, repeated measures ANOVA showed a significant group X task interaction [F(1,24) = 6.51; p = 0.018]; post hoc analysis showed that absolute timing error in the human body motion task was greater at all target intervals in the 1Hz-rTMS group compared with the Sham group (p = 0.021), whereas there was no difference between groups on the absolute timing error in the inanimate object motion task (p = 0.46). Moreover, although subjects in the Sham group exhibited a similar absolute timing error in both tasks (p = 0.36), subjects pre-treated with 1Hz-rTMS exhibited a greater absolute timing error in the human body motion task compared with the inanimate object motion task (p = 0.015). A significant main effect for target interval [F(2,48) = 29.94; p<0.0001] was also observed, and post hoc analysis showed that the absolute timing error was greater for the shortest dark intervals compared with the longer ones in both groups (6 versus 9 s and 12 s: p<0.0001). No major effect of task or group or group X task X target interval interaction was observed (p> 0.05).

**Fig 3:**
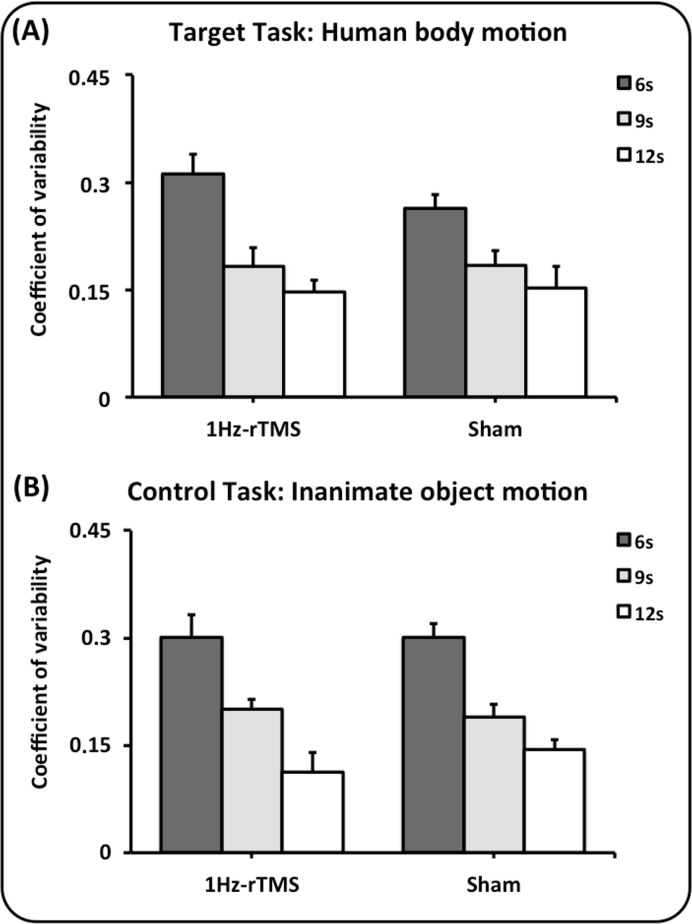
Effect of 1Hz-rTMS and Sham rTMS on lateral cerebellum on the coefficient of variability calculated as standard deviation/mean of the reproduced intervals. Abscissa: the type of conditioning stimulation (1Hz-rTMS and Sham). Ordinate: the value of the coefficient of variability. Panel A depicts the results of the human body motion perceptual task, whereas panel B depicts the results of the inanimate object motion task. Colours code the duration of the dark interval: dark grey, 6 seconds; light grey, 9 seconds; white, 12 seconds. Mean data + standard error mean (SEM) are shown.

Variability data are reported in Fig. 3. Repeated measures ANOVA on coefficient of variability data showed a significant main effect for target interval [F(2,48) = 72.20, p<0.0001]. Timing performance was proportionally more variable when target intervals were shorter (6 versus 9 s and 12 s: p<0.0001; 9 versus 12 s: p<0.0001). Conversely, there was no difference between groups, as we did not observe any significant main effect of either group or group x task, group x target interval and group x task x target interval interaction terms (all P > 0.05).

Timing error data are reported in Table 1. Timing error was influenced by the nature of the task and the duration of the dark interval. Indeed, a greater shift towards the over-estimation (expressed as a timing error positive value) of the dark interval was observed in the human body motion task (handwriting) compared with the inanimate object motion task, in both groups and at all the dark intervals evaluated (task: F(1,24) = 6.61; p = 0.017). Further, we observe a global tendency of subjects to over-estimate the dark interval when this was shorter (6 seconds) and to under-estimate it when this was longer (12 seconds) (timing error negative value): RM-ANOVA showed a main effect of target interval (F(2,48) = 98.70; p< 0.001), and post hoc analyses showed that when the dark interval was 6 seconds timing error was significantly more positive than for either 9 and 12 seconds dark interval durations (6 versus 9 s and 12 s: p<0.0001; 9 versus 12 s: p<0.0001). Inhibitory rTMS on lateral cerebellum did not modify these results and no difference between groups was found (main effects of group and interaction group x task, group x target interval and group x task x target interval always not significant, p>0.05).

**Table 1:** Value of timing error in milliseconds in the target task and in the control task of both the 1Hz-rTMS group and the Sham group (mean ± sd).

	Target Task: human body motion			Control task: inanimate object motion		
Dark Interval	6s	9s	12s	6s	9s	12s
1Hz-rTMS	2318.2±2182	396.4±2900	-1081.2±3274	1474.9±960	-341.5±1600	-1881.9±2300
Sham	1927.2±1766	377.1±1215	-536.5±1661	1028.1±130	-552.6±1900	-2387.9±1700

**Fig 4:**
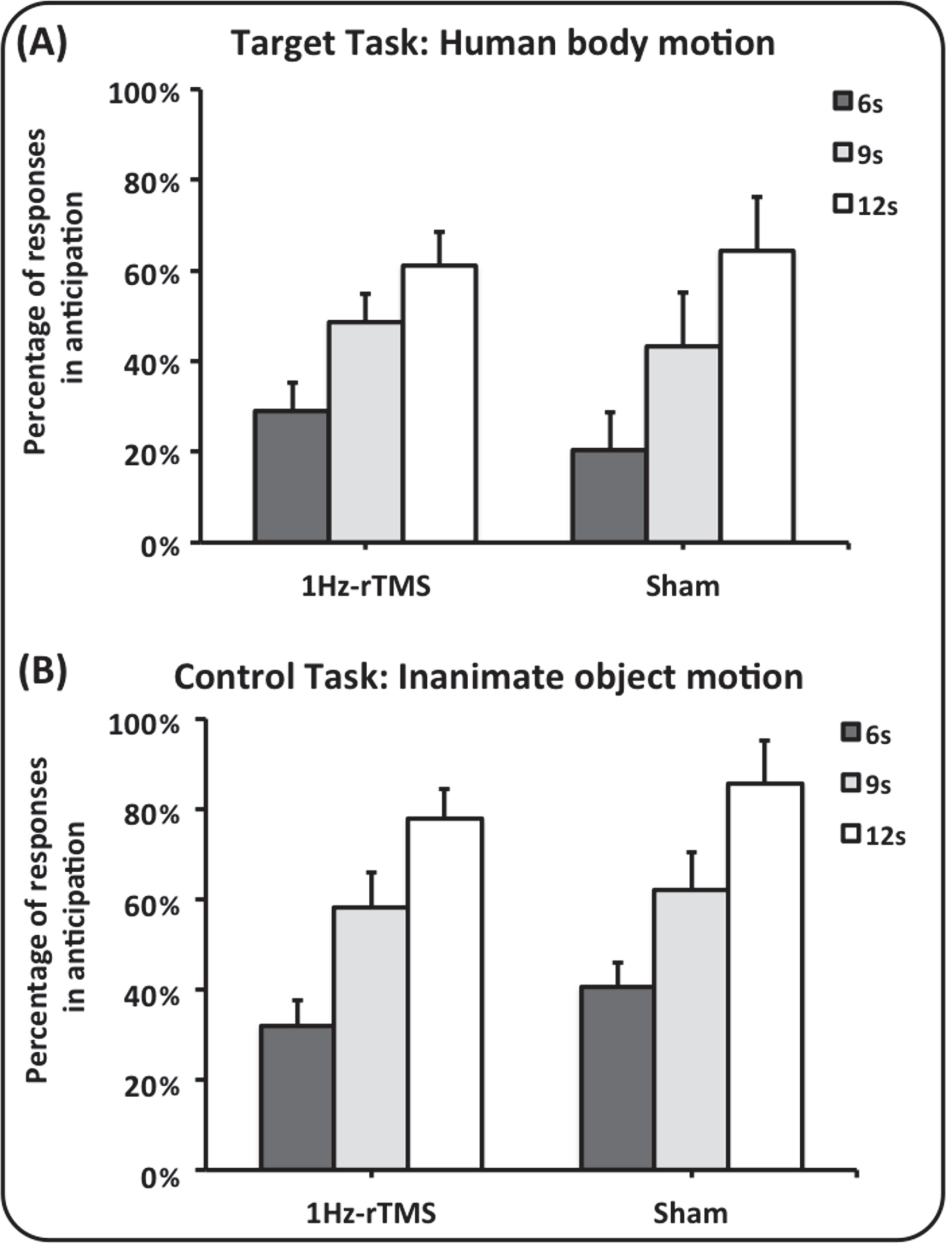
Effect of 1Hz-rTMS and Sham rTMS on lateral cerebellum on the percentage of responses in anticipation. Abscissa: the type of conditioning stimulation (1Hz-rTMS and Sham). Ordinate: the percentage of the response in anticipation with respect to the total number of trials. Panel A depicts the results of the human body motion perceptual task, whereas panel B depicts the results of the inanimate object motion task. Colours code the duration of the dark interval: dark grey, 6 seconds; light grey, 9 seconds; white, 12 seconds. Mean data + standard error mean (SEM) are shown.

The results on percentage of responses in anticipation paralleled those on timing error. The percentages of response in anticipation for both target and control tasks are reported in Fig. 4. Repeated measures ANOVA showed a significant main effect for task [F(1,24) = 6.27, p = 0.019] and target interval [F(2,48) = 45.44, p<0.0001]. Both groups of subjects significantly underestimated more target intervals in the inanimate object motion task than in the human body motion task (P = 0.019). Further, both groups tended to underestimate more the duration of the target interval when it was longer (9 and 12 s) (6 versus 9 s and 12 s: p<0.0001; 9 versus 12 s: p<0.0001). No difference between groups was found (main effects of group and interaction group x task, group x target interval and group x task x target interval always not significant, P >0.05).

## Discussion

In the present study we showed that the inhibition of lateral cerebellum activity, by means of 1Hz-rTMS, induced a worsening in timing performance when participants were engaged in a perceptual temporal expectation in which they had to make a deliberate estimate of the duration of a perceived common movement of a body segment (i.e. handwriting). Differently, we did not observe any effect of 1Hz-rTMS when subjects had to predict the outcome of a movement involving an inanimate object (i.e. a ball reaching a target).

Confirming our previous observations, apart from a specific effect of inhibitory 1Hz-rTMS, the ability to estimate the time point at which the visually perceived movement ended was influenced by the duration of the time interval in which subjects had to rely only on their mental representation of movement (i.e. the dark interval) and by the type of motion perceived.

Shorter dark intervals were associated with higher tendency to shift the end of the perceived movement ahead in time (i.e., a more positive timing error), with greater variability of prediction response and greater normalized absolute timing error. These results, in accordance with what observed in a previous work of our group [9], lend support to the reproducibility of our temporal expectation task across different groups of subjects [9]. It has already been described in the literature that when humans are asked to reproduce various temporal intervals, shorter durations are perceived as being longer than the reference, and the opposite is true for long durations. This phenomenon of regression to the mean is known as Vierordt’s law [23], and it has also been found in studies of ‘pure’ temporal reproduction [24]. The normalized absolute timing error and the coefficient of variability are both approximate ratios of the target interval duration, thus allowing for comparison of performance at each interval independent of duration [25]. The effect of the target interval on these two parameters is, however, not surprising given that both tend to be larger for shorter target intervals compared with longer ones [25–28].

Further, higher frequency of anticipated responses and a more negative timing error were observed on the inanimate object motion task compared with the human body motion task. Again, this finding is in accordance with what observed in a previous work of our group [9], and is consistent with data present in the literature suggesting that the ability to predict the temporal outcome of a perceived action changes depending on whether the action is performed by a body segment (e.g. a limb) or an inanimate object [29].

### The role of cerebellum in temporal expectation

Temporal expectations make use of timing information in order to optimise motor or perceptual performance [8]. For example, to perceptually estimate whether a car might cross one’s way and to react in time, the sensorimotor system would benefit from predicting the car’s upcoming movement based on sensory input and constantly recalibrating sensory predictions to match the ever-changing properties of one’s sensorimotor system and environment. The same proof of concept can be applied in the temporal estimation of a human motor act. Let’s think about a tennis player, who has to predict the end of his opponent’s tennis shot in order to react in time.

In agreement with the role of cerebellum in processing and adapting motor related information, it has been shown that the cerebellum is needed to optimize self-action by recalibrating predictions capturing the sensory consequences of one’s actions [30–32]. However, the cerebellum seems also implicated in predictions exclusively related to a perceptual domain. Indeed, when subjects used temporal information inherent the spatial-temporal trajectory of a dynamic visual stimulus to predict its final position, activations were reported in left lateralized inferior parietal cortex [10], sensorimotor regions of premotor and parietal cortices [11], and cerebellum [12].

In the present study, we wanted to expand this topic by exploring the role of cerebellum in predictions related to a visually perceived motor act (here, handwriting). We found that subjects’ precision in in temporally predicting the end of a human movement (referred here to the absolute value of timing error) was influenced by 1Hz-rTMS disruption, whereas the variability in temporal expectation performance and the direction of the timing error (expressed by both the timing error and the percentage of responses in advance) were not influenced by 1Hz-rTMS disruption.

In self-action it is well known that cerebellum is involved in predictive control of movement [33]. The term “predictive” refers to the feed-forward portion of movement that is planned in advance and is unchanged by online peripheral feedback during motor execution. Forward models capture the expected sensory consequences of one’s actions based on current body state and the efference copies of motor commands [33] and enable fast and accurate movements despite delayed or missing sensory feedback [34–37].

Here, we demonstrated for the first time that cerebellum is involved in predictive movement control in absence of self-action; i.e., during observation of a motor act, thus confirming recent theories supporting a cognitive-perceptual role of the cerebellum [38]. In accordance, it has been suggested that the cerebellum is part of the human action observation network [39–42] mediating the interactions of self-executed movements in biological motion perception (i.e., action-perception coupling) [43].

The perceptual predictive movement control operated by the cerebellum might be implicated in motor planning and its disruption might contribute to some movement abnormalities exhibited by patients with cerebellar damage, like lack of coordination, and poor accuracy [44]. However to what extent the prediction of temporal outcome of observed motor actions operated by the cerebellum may affect motor execution has to be determined in future studies.

Intriguingly, when cerebellar activity was disrupted by inhibitory 1Hz-rTMS we did not observe any modification in time estimation when the perceived movement was that of a moving object (not a biological motion). Although this does not exclude a cerebellar involvement in predicting the outcome of a moving object [12], it nevertheless seems consistent with previous studies suggesting cerebellar activation during the encoding of a biological motion with respect to other stimuli [29,42,45]. This suggests that cerebellar structures are specifically involved in processing the temporal aspects of a movement belonging to the human repertoire thus probably making this task more sensible to rTMS disruption.

In addition, there are some methodological characteristics of the study conducted by O’Reilly and co-workers that could partially justify this discrepancy, like the presence of an occluder, the type of task (temporo-spatial instead of temporal only), the type of participants response (a qualitative dycotomic one instead of a quantitative one) [12].

Another noteworthy aspect of our results is that cerebellar disruption led to an impact on supra-second intervals. Most studies available in the literature suggest that the cerebellum is involved in sub-second timing, although some lesion studies suggest that the cerebellum is also involved in supra-second intervals [25]. Notably, two studies, using repetitive transcranial magnetic stimulation, determined that the cerebellum is essential in timing in the range of millisecond time intervals and not of supra-second time intervals [16,46]. However, these studies analysed the role of cerebellum in explicit motor and perceptual timing, whereas our study focused on temporal expectation, i.e., an implicit perceptual timing task, likely engaging a different cerebral network [47]. In addition, to our knowledge this is the first study that directly analyse the role of cerebellum in time processing of a motor act, whereas previous studies using rTMS adopted tasks requiring either motor production or perceptual discrimination of a timed duration [16,46].

It’s worthy to note that although we demonstrated that lateral cerebellum is directly involved in a temporal expectation task whose goal is to predict the temporal outcome of a motor act, we can only do some speculation about the cortical areas that may be recruited in this perceptual-motor ability. Indeed, given the cerebellar connectivity with cortical regions engaged in higher-order perceptual processing and spatiotemporal awareness (e.g., posterior parietal and premotor cortex) [48,49], we may speculate that a cerebral network comprising premotor cortex, parietal cortex and the cerebellum could guarantee an adequate timing prediction. However, the precise definition of the cerebral network engaged in everyday temporal expectations related to human motor acts needs to be elucidated in future studies.

One might argue that changes in motor performance, induced by cerebellar 1Hz-rTMS might have influenced the performance on the temporal expectation task. When applied to lateral cerebellum, 1Hz-rTMS is known to induce a disruption of cerebellar outputs to the contralateral motor cortex (M1). Low-frequency right cerebellar stimulation has been demonstrated to induce a decrease in the excitability of intracortical facilitatory circuits in left M1, likely due to the reduction of excitatory drive of cerebellar nuclei to the motor cortex, thus reflecting a suppressive effect of low-frequency rTMS on cerebellar Purkinje cells [20]. Further, low-frequency right cerebellar stimulation can influence motor performance [17]. Since our subjects were asked to press the space button with their right hand to make their responses, the observed modifications in the execution of the temporal prediction task might have been caused by a modification in the left M1 excitability and/or right hand performance. However, the impairment in temporal prediction selectively in the target task (handwriting) and not in the control one (moving ball) cannot be attributed to an effect of 1Hz-rTMS on motor function because identical motor responses were required for the two conditions.

## Conclusions

We propose that the cerebellum is directly implicated in temporal expectation, when timing information is processed in order to make accurate predictions on the outcome of an observed motor act. This result enlarges the current view of the cerebellum as a “non-motor” cerebral structure, engaged in cognitive and perceptual domains. However, the higher susceptibility of cerebellar activity to 1Hz-rTMS disruption for time estimation of a human motor act, rather than that of a moving object (not a biological motion), suggests that cognitive and perceptual functions of cerebellum are likely to be strictly connected to motor control.
